# Dynamic Changes in Central and Peripheral Neuro-Injury vs. Neuroprotective Serum Markers in COVID-19 Are Modulated by Different Types of Anti-Viral Treatments but Do Not Affect the Incidence of Late and Early Strokes

**DOI:** 10.3390/biomedicines9121791

**Published:** 2021-11-29

**Authors:** Krzysztof Laudanski, Jihane Hajj, Mariana Restrepo, Kumal Siddiq, Tony Okeke, Daniel J. Rader

**Affiliations:** 1 The Department of Anesthesiology and Critical Care Medicine, University of Pennsylvania, Philadelphia, PA 19104, USA; 2 Department of Neurology, University of Pennsylvania, Philadelphia, PA 19104, USA; 3 The Leonard Davis Institute of Health Economics, University of Pennsylvania, Philadelphia, PA 19104, USA; 4 School of Nursing, Widener University, Philadelphia, PA 19013, USA; jjhajj@widener.edu; 5 College of Arts and Sciences, University of Pennsylvania, Philadelphia, PA 19104, USA; rmariana@sas.upenn.edu; 6 College of Arts and Sciences, Drexel University, Philadelphia, PA 19104, USA; ks3743@drexel.edu; 7 School of Biomedical Engineering, Drexel University, Philadelphia, PA 19104, USA; tko35@drexel.edu; 8 Department of Genetics, University of Pennsylvania, Philadelphia, PA 19104, USA; rader@pennmedicine.upenn.edu

**Keywords:** COVID-19, neurodegeneration, neuroinflammation, clusterin, fetuin, CCL23, remdesivir, convalescent plasma, steroids, stroke

## Abstract

The balance between neurodegeneration, neuroinflammation, neuroprotection, and COVID-19-directed therapy may underly the heterogeneity of SARS-CoV-2′s neurological outcomes. A total of 105 patients hospitalized with a diagnosis of COVID-19 had serum collected over a 6 month period to assess neuroinflammatory (MIF, CCL23, MCP-1), neuro-injury (NFL, NCAM-1), neurodegenerative (KLK6, τ, phospho τ, amyloids, TDP43, YKL40), and neuroprotective (clusterin, fetuin, TREM-2) proteins. These were compared to markers of nonspecific inflammatory responses (IL-6, D-dimer, CRP) and of the overall viral burden (spike protein). Data regarding treatment (steroids, convalescent plasma, remdasavir), pre-existing conditions, and incidences of strokes were collected. Amyloid β42, TDP43, NF-L, and KLK6 serum levels declined 2–3 days post-admission, yet recovered to admission baseline levels by 7 days. YKL-40 and NCAM-1 levels remained elevated over time, with clusters of differential responses identified among TREM-2, TDP43, and YKL40. Fetuin was elevated after the onset of COVID-19 while TREM-2 initially declined before significantly increasing over time. MIF serum level was increased 3–7 days after admission. Ferritin correlated with TDP-43 and KLK6. No treatment with remdesivir coincided with elevations in Amyloid-β40. A lack of convalescent plasma resulted in increased NCAM-1 and total tau, and steroidal treatments did not significantly affect any markers. A total of 11 incidences of stroke were registered up to six months after initial admission for COVID-19. Elevated D-dimer, platelet counts, IL-6, and leukopenia were observed. Variable MIF serum levels differentiated patients with CVA from those who did not have a stroke during the acute phase of COVID-19. This study demonstrated concomitant and opposite changes in neurodegenerative and neuroprotective markers persisting well into recovery.

## 1. Introduction

Severe acute respiratory syndrome coronavirus 2 (SARS-CoV-2) has been linked to abnormalities in the central nervous system such as the loss of smell and taste, cognitive decline, delirium, cerebrovascular accidents, neuronal autoimmune illnesses, and psychiatric disorders [1,2,3,4,5,6,7]. However, the heterogeneity of symptoms remains puzzling [1,3,7,8,9,10]. The development of these abnormalities often occurs within two days from the onset of symptoms [1,5,7,11,12]. In addition, an increasing number of survivors suffer from long-term neurological sequela [13,14,15,16].

Coronaviruses contribute to the emergence of neuropathologies via several mechanisms: direct neurotoxic effects to homogenous and retrosynaptic transport, localized neuroinflammation, blood–brain barrier (BBB) dysfunction, and secondary nervous tissue damage in response to generalized inflammation and hypoxia [7,8,17,18,19,20]. Moreover, SARS-CoV-2 co-localizes with the phosphorylated tau (τ) protein, triggering neuronal death or interfering with the distribution of τ [21]. The latter effect persists, suggesting a potential chronic mechanism for neurodegeneration. The spike protein S1 subunit (S1) receptor-binding domain binds to several neuronal proteins, including Amyloid-β1, α-synuclein, τ, prion, and TAR DNA-binding protein 43 (TDP-43) [22]. Amyloid β1-42, but not Aβ1-40, exhibits a preferentially high affinity for the S1 protein of SARS-CoV-2 and angiotensin-converting enzyme 2 (ACE2) [23]. N-protein in silico interacts with RNA and is key for the formation of stress granules in the host [24]. This interference with several chaperone proteins is a hallmark of coronavirus proteins [25]. These interactions can trigger the aggregation of the brain’s proteins when protective anti-folding mechanisms are impaired, potentially exacerbating ongoing neurodegeneration processes and effects of the non-specific inflammatory response [26,27,28,29,30]. Several case reports, case series, and research projects provided conflicting data regarding the levels of τ, Amyloid β1-42, and Amyloid β2-41 in COVID-19 [6,31,32,33,34,35]. In silica, analyses suggested molecular mimicry between the compromising of SARS-CoV-2 and nervous system proteins, but clinical correlations are not described [3,36].

In a “classical” neurodegeneration illness, Alzheimer’s disease (AD), τ protein, phosphoτ181, and amyloids (β40 and β42) are critical modulators, and their burden correlates with disease severity [37,38,39,40,41]. Neurogranin (NRG), TAR DNA-binding protein 43 (TDP43), chitinase 3-like 1 (YKL40, cartilage glycoprotein-39), and kallikrein 6 (KLK6) are often found in classical AD and other dementias [42,43,44,45,46,47,48,49]. Markers of neuronal (neurofilament light, NF-L) and glial fibrillary acidic protein (GFAP) are commonly elevated in neurodegenerative illnesses due to underlying neuron injury and secondary to immune system activation, hypoxia, and high free radical environment [41,42,50]. This somewhat complex landscape indicates the heterogeneity of the dementias and cognitive decline in general. It also raises the question of how the heterogeneity of COVID-19 neurological presentations reflects the diverse ways SARS-CoV-2 interacts with neuronal systems instead of one particular disease [1,9,10]. Several biomarkers of neurological dysfunction have been reported as altered in neurodegenerative cognitive disorders, psychosis, schizophrenia, and neurodysfunctional processes: ailments commonly found in COVID-19 patients [51,52,53,54,55]. Furthermore, rather than their absolute levels, the dynamics of markers are more important for predicting cognitive outcomes than isolated measurements [56,57]. However, only a few of these markers were studied in COVID-19, but even fewer look at them longitudinally in COVID-19 survivors [12,34].

Apart from direct SARS-CoV-2 neurotoxicity, general and local immune activation can accelerate neurodegeneration and neuro-injury [58]. Microglia, astrocytes, and oligodendrocytes provide a local environment governing neuroinflammation trajectories and critically impact the trajectory of the neurodegeneration process and subsequent outcomes [59,60,61,62,63,64]. Furthermore, the influx of peripheral leukocytes into the central nervous system via a dysfunctional BBB can fuel neuroinflammation [65,66,67]. Monocyte chemoattractant proteins (MCP), monocyte inhibitory factor (MIF), C-X-C motif ligand 13 (CXCL13), and C-C motif ligand 23 (CCL23), have been implicated in intraparenchymal leukocyte migration, vasculitis and, in the case of CCL23, MIF, and MCP-1, are linked to the progression of dementia by supporting chronic neuroinflammation [68,69,70,71,72,73,74]. Concomitantly, the radiographic indicators of neuronal damage and vascular injury persist for three months after the resolution of COVID-19 [75,76,77]. Considering the high degree of inflammation, it is not surprising that cerebrovascular events are relatively common in COVID-19 along with some cognitive decline [11,78,79]. Furthermore, CCL23 and MCPs have been connected to the severity of the clinical presentation of COVID-19 while being linked to the emergence of chronic neurodegeneration under other circumstances [80,81]. This post-COVID-19 inflammation may be particularly severe in individuals with pre-existing cognitive vulnerability [82,83,84,85,86,87]. Furthermore, a relative excess of cerebrovascular events in COVID-19 may exacerbate the cognitive decline in primary or secondary neurodegenerative illnesses [11,78,79]. Finally, unfavorable pre-existing homeostasis (lack of antioxidants, nutrition deficiency, others) may increase the degenerative potential of COVID-19-driven inflammation [88,89,90]. Excessive burden from pre-existing diseases and malnutrition are commonly seen in the populations most-stricken by COVID-19. Poor nutritional status may be of particular importance.

Neurodegenerative and neuro-injury processes are counterbalanced by several protective mechanisms such as limiting excessive damage by immune system activation, complement proteins, free radicals, and other mechanisms [86,91,92,93]. Clusterin, fetuin-A, and TREM-2 are examples of biomarkers linked to delays in the progression of neurodegeneration [86,91,92,93]. The depletion of these factors results in the progression of neurodegenerative disorders and excess cerebrovascular strokes [85,86,91,92,93,94,95,96,97]. Apolipoprotein E (apoE) was frequently linked to dementia progression in general, but in COVID-19, this factor may be of particular importance [98]. However, there is a gap in knowing how the neuroprotective mediators counterbalance the neurodegeneration processes in COVID-19 [12]. Interestingly, implementation of several anti-COVID-19 therapies should, directly and indirectly, affect COVID-19 related neurodysfunction. However, it remains unclear how SARS-CoV-2-related therapies affect COVID-19-associated neuroinflammation and neurodegeneration [10,87,99]. Hypothetically, by decreasing the burden of the pathogens (remdesivir), and by modulating the immune system response (steroids, convalescent plasma), they should have a positive effect [100,101,102].

The purpose of this longitudinal study is to examine the long-term dynamics of biomarkers of neuroinflammation, neurodegeneration, and neuroprotective milieu in hospitalized patients with COVID-19 while considering the timing of the illness and immunological response in the context of stroke occurrence. We hypothesize that the elevation of neuronal degeneration markers would resolve over time in survivors, particularly in individuals treated with COVID-19-related therapy. We hypothesize that increases in neuroinflammation markers can be linked to the incidence of strokes during our follow-up windows. Most importantly, we investigate if the initial increase in neurodegeneration markers will be accompanied by a decline in neuroprotective proteins, creating conditions that could promote the release of neurodysfunctional markers and their potential long-term outcomes.

## 2. Materials and Methods

### 2.1. Patient Enrollment

Our study protocol was approved by the Institutional Review Boards (IRB) of the University of Pennsylvania and was performed according to the ethical guidelines of the 2003 Helsinki Declaration (#813913; approved 3 February 2020). Patients admitted to the hospital between March 2020 and December 2020 with a diagnosis of COVID-19, via PCR confirmation, were approached for written consent.

Upon consent, blood was collected in vacutainer tubes with heparin, cooled, and spun down. Serum was isolated and stored at −80 °C. In order to anchor the samples to common, relative timepoints in the progression of patients’ COVID-19, all samples were divided as admission (drawn within 48 h of admission), H2 (+3–4 days after admission), and H3 (+4–7days) (Supplemental Figure S1). In addition, H4 samples were collected from inpatient and outpatients samples between 8 days and 28 days if the patient was available during a routine visit to the healthcare system to provide a convenience sample. 

### 2.2. Clinical Data

The electronic medical records (EMR) was used to collect the demographic and clinical data for all the enrolled participants. Patients self-determined their race and ethnicity. The Acute Physiology and Chronic Health Evaluation II (APACHE II) Score was calculated within one hour (APACHE_1_) and at 24 h after admission (APACHE_24h_) [103,104]. The burden of chronic disease was calculated using the Charlson’s Comorbidity Index (CCI) [105]. The severity of illness was determined via the Marshalls Organ Dysfunction Score (MODS) [106]. Survival was determined at six months from admission. The incidence and nature of cerebrovascular events were determined from medical records. Long-term follow-ups extended up to 180 days, depending on the availability of patient records. If no records were available at that time, we assumed that patients did not expire and did not experience a CVA.

The information on treatments with remdesivir, convalescent plasma, and steroids was extracted from medical records. Except for the latter, the treatments were highly protocolized per hospital policy and according to the FDA recommendations for the given treatment. Steroid treatment was defined as the engagement of any intravenous or oral glucocorticoid steroid compounds to treat COVID-19 pneumonia per the healthcare provider’s notes.

### 2.3. Assessment of Biomarkers

Neurological (τ, phospho τ, amyloid β-40, amyloid β-42, TDP43, NRGN, YKL40, NCAM-1, KLK6, clusterin, and fetuin), cytokine (MIF, IL-6, TNF, MIP), and inflammatory (D-dimers, ferritin) markers were measured using a multiplex kit (Theromofisher, Waltham, MA, USA) on a MagPix machine (Luminex; Austin, TX, USA). In addition, commercial enzyme-linked immunoassays were used to measure NF-L (US Diagnostic, Boston, MA, USA), CCL23 (R&D), and MCP-1 (Biolegend, San Diego, CA, USA). Per protocol, all samples were inactivated with (5%) Triton X-100 (ChemCruz, Dallas, TX, USA).

### 2.4. Assessment of SARS-CoV-2 Disease Burden

The level of S-protein was measured using commercially available kits (RayBiotech, Stanford, CA, USA). The levels of specific immunoglobulins against proteins S and N were measured using commercially available kit (RayBiotech, Stanford, CA, USA). The absorbance OD values were subtracted from albumin-coated plates and referenced against the standard curve.

### 2.5. Statistical Analysis

The Shapiro–Wilk W test and distribution plots were used to test the normality of distribution variables. Parametric variables were expressed as mean ± SD and compared using Student’s *t*-test. For non-parametric variables, median (Me) and interquartile ranges (IR) will be shown with the U-Mann–Whitney statistic employed to compare such variables. ANOVA was calculated for parametric variables with multiple discrete values with Shaffer’s test as a post hoc test. Correlation momentums were calculated as *r*^2^ Pearson values with *r*^2^ more than 0.25 being significant correlations. The regression analysis was completed using stepwise methods. A *p*-value of less than 0.05 was considered statistically significant for all tests. Statistical analyses were performed with SPSS 26 (IBM, Waltham, NY, USA). 

## 3. Results

### 3.1. Characteristics of the Study Cohort

COVID-19 patients were predominantly Black (66.7%) and male (60.0%), with the average age being 58.9 + 14.61 (X ± SD) years old (Table 1). Patient dispositions were similar at one and six months, with an increasing number of patients deceased at six months (Supplementary Figure S2). There was no significant difference between our initial cohort in terms of demographic and clinical data, except the length of stay was significantly longer in the cohort that participated in the 6-month follow-up (Table 1).

**Figure 1:**
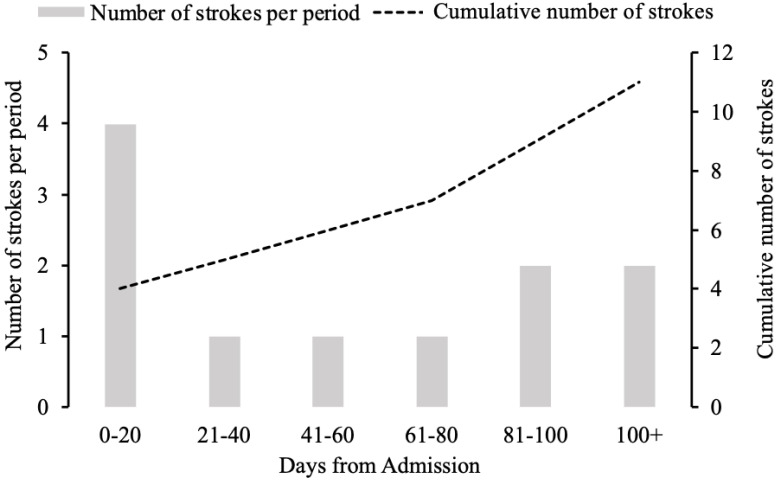
The distribution of cerebrovascular accidents that occurred following hospital admission.

Five patients were admitted with stroke as one of their principal diagnoses. Between 28 days and six months, an additional six strokes occurred (2.9% ischemic stroke, 2.9% hemorrhagic stroke, and 0% mixed nature) (Figure 1). Around 9.5% of patients had a history of pre-existing stroke, but only one had an acute stroke following COVID-19 hospitalization in that group. One patient suffered two strokes during the observation period. The APACHE scores at 6 months and MODS scores at 48 h from admission were significantly increased among patients with a cerebrovascular event more than patients without a CVA (Table 1). However, mortality was similar between patients with or without CVA events (Table 1).

Patients with new cerebrovascular events after admission had similar burdens of comorbidities measured (CCI_Recovered_ = 3.5 + 3.40 vs. CCI_CVA_ = 5.1 + 3.40; t [33;18] = 0.357, *p* = 0.553) (Table 1). However, the pre-existing diseases of patients with stroke vs. no stroke indicated that histories of congestive heart failure (χ^2^ = 8152; *p* = 0.043) and AIDS (χ^2^ = 34.12; *p* = 0.0001) were more common in patients with early (less than 28 days), rather than late strokes (less than 6 months) (data not shown). The incidence of pre-existing stroke in patients suffering from a cerebrovascular incident during COVID-19 hospitalization vs. patients without CVA during that time were comparable (10.0% vs. 11.3%, respectively).

Mortality in the studied cohort was 21.9%, with some patients dying within 48 h from admission (Table 1; Supplemental Figure S2). Mortality among patients with stroke was 40%, and 50% of these deaths occurred 110 days following admission. Patients with stroke had a higher, statistically insignificant admittance rate to the ICU (Table 1).

### 3.2. The Dynamics of Neurodegeneration and Neuroinflammation after COVID-19 and Their Relationship to Inflammation and Viral Burden

**Figure 2:**
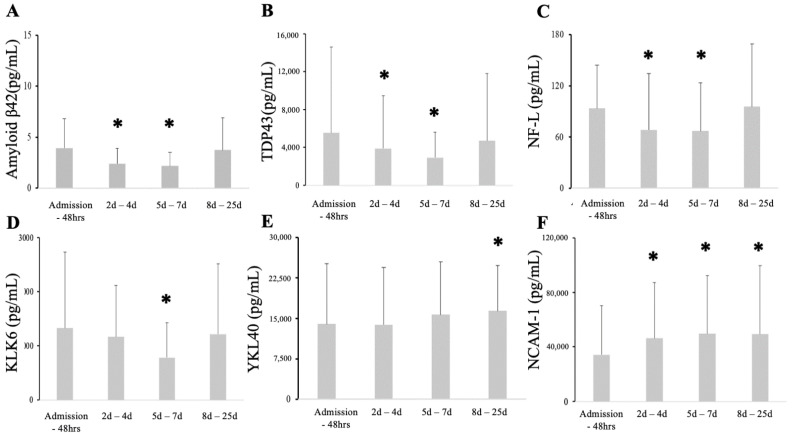
Amyloid β42, TDP43, and NFL serum levels diminished shortly after admission to recover at 28 days (**A**–**C**). KLK6 decreased significantly at seven days, then recovered to baseline levels (**D**). Serum YKL40 increased in sample taken more than 7 days after admission (**E**). NCAM-1 significantly and sustainably increased afteno, it should pr admission (**F**). * Statistical difference when compared to all patients at admission.

The serum levels of neuro-injury and neurodegeneration markers followed three time patterns. Amyloid-β42, TDP43, and NF-L significantly declined at H2 and H3 compared to initial levels (H1) and rebounded at H4 (Figure 2A–C). KLK6 levels declined at H3 (Figure 2D). YKL-40 was statistically elevated at H4, while NCAM-1 was elevated at all post-admission time points (Figure 2E,F). There were no differences in serum levels of Amyloid-β40 for any measured time points (data not shown). Levels of total τ protein and phosphorylated τ were highly variable, with few individuals having significant increases in these serum levels while the majority were below detectable levels (Supplementary Figure S3). However, serum levels of the S-protein correlated with phospho τ (*r*^2^ = 0.25; *p* < 0.000001) (Supplementary Figure S4). Concomitantly, TDP-43 correlated highly with ferritin at admission (*r*^2^ = 0.73; *p* < 0.00001) and during the remainder of the hospitalization (*r*^2^ = 0.39; *p* < 0.000001) (Supplementary Figure S4). Similarly, KLK6 correlated highly with ferritin at admission (*r*^2^ = 0.5; *p* < 0.00001) and weakly throughout the hospitalization (*r*^2^ = 0.24; *p* < 0.000001) (Supplementary Figure S4). Other correlations were weak yet statistically significant (Supplementary Figure S4). There was no significant correlation between platelets, WBC, and D-dimer (Supplementary Figure S4).

**Figure 3:**
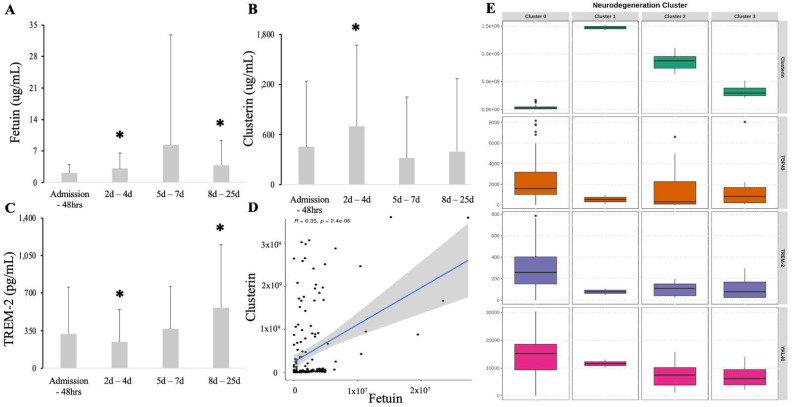
Serum levels of fetuin (**A**), clusterin (**B**), and TREM-2 (**C**) compared to admission. The correlation between fetuin and clusterin serum levels (**D**). Clusterin analysis revealed 4 clusters (**E**), one with depletion of clusterin (#0) while the clusters #1, #2, #3 are signified by depletion of TREM-2. * Statistical difference when compared to all patients at admission (*p* < 0.05).

Fetuin serum levels increased at 48 h and 7 days from admission yet were variable (Figure 3A). Clusterin levels were significantly elevated immediately after admission (H2), followed by a return to baseline levels at the 3 day mark (Figure 3B). Contrary to fetuin and clusterin, TREM-2 levels were depleted at 48 h but significantly elevated by 28 days (Figure 3C). Both fetuin and clusterin correlated highly with each other (*r*^2^ = 0.68; *p* = 0.00001) (Figure 3D). A cluster analysis, including neurodegeneration and neuroprotective markers, revealed four clusters (Figure 3E). Cluster #0 demonstrated a significant depletion of clusterin with concomitant elevations in YKl-40 and TDP43 and preserved TREM-2 (Figure 3E). Clusters #1, #2, and #3 displayed decreases in TREM-2 while clusterin levels were preserved (Figure 3E). Fetuin and other neurodegeneration markers (τ, phospho τ, amyloids, NCAM-1) were not significant factors in creating clusters (data not shown).

### 3.3. The Dynamics of Neurovasculitis Markers during COVID-19

The trajectory of neuroinflammatory markers reflected elevated CCL23 serum levels for all patients at H4 (Table 2). In addition, MCP levels and MIF levels returned to near-admission levels following a significant decline at 7 days from admission (Table 2).

### 3.4. Effect of COVID-19 Directed Therapies on Neurodegeneration, Neuroinflammation, and Inflammatory Markers

**Figure 4:**
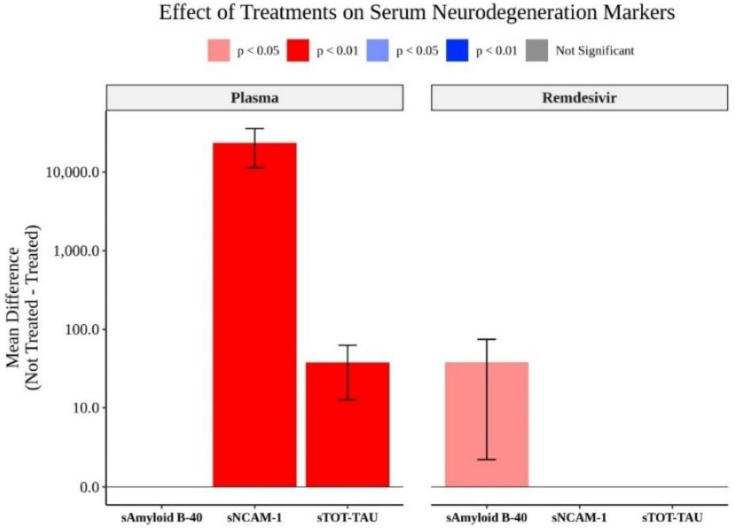
The effects of convalescent plasma, remdesivir were limited to total τ, amyloid β40, and NCAM-1.

Patients not treated with convalescent plasma demonstrated significantly higher levels of NCAM-1 and total τ (Figure 4). Patients not treated with remdesivir had significantly elevated levels of Amyloid-β40 (Figure 4). The steroid treatment did not significantly alter any markers measured (data not shown).

### 3.5. Relationship between Stroke and Clinical Markers

There was no statistical difference between the initial severity of clinical presentation in patients with and without stroke as measured by APACHE at one hour from admission, yet the scores at 24 h were greater for stroke patients (Table 1). In addition, initial MODS and SOFA scores for the first 48 h remained similar for all patients and became significantly worse when looking at patients with stroke (Table 1; Supplemental Table S1). Platelet levels increased significantly at H2 for all patients and patients who did not experience a stroke, followed by a recovery at H4 (Table 2). Elevated WBC counts were seen in all patients, on average, at more than 3 days following admission, with stroke patients having significantly higher levels than the non-stroke group at H4 (Table 2). Clinically, COVID-19 patients diagnosed with acute stroke exhibited higher serum IL-6 than patients not diagnosed with acute stroke, starting at 3 days post-admission (Table 2). This cohort of patients also demonstrated significantly decreased serum IL-8 48 h after admission, yet recovered to admission levels after 7 days (Table 2). In addition, TNFα levels slightly increased before returning to baseline levels (Table 2) There were also no significant differences for the procalcitonin serum levels of stroke patients, although they did exhibit significantly higher D-dimer levels at H4 than those not diagnosed with acute stroke (Supplemental Table S1). Finally CRP (ΔXdifference = +1.03 CI: 0.057–2.04; *p* = 0.038) and τ (ΔXdifference = +3.27 CI: 3.31–6.21; *p* = 0.029) were elevated in patients with stroke in the wake of COVID-19 (data not shown).

## 4. Discussion

This is the first study showing dynamic changes in neurodegeneration and neuroprotective markers in COVID-19 patients. We found that several neurodegeneration markers (YKL40, NCAM-1, CCL23) were elevated in survivors of COVID-19 in a sustained fashion. Others (Amyloid β42, KLK6, TDP43, NF-L) declined in the intermittent period after admission before returning to their original levels in survivors. Contrary to this, neuroprotective factors (fetuin, clusterin, TREM-2) were highly variable across the observed time periods. Thus, our study suggests an emergence of imbalance strongly favoring the neurodegeneration process in individuals with acute COVID-19, a suggestion that was theorized but not clinically examined [1,9,10,13,14,87].

The sustained elevation of YKL40, NCAM-1, and CCL23 may represent an ongoing pathological process in the survivors of COVID-19 as the levels did not decline below those recorded at admission [46,51,107]. These bio-spectrum markers are not specific to one type of dementia but rather represent the beginning or acceleration of underlying neurodegeneration processes in the wake of COVID-19 [1,13,108,109]. This finding is consistent with the suggestion of ongoing brain injury in post-viral syndromes or COVID-19 long-haulers [10,13]. TDP43 was particularly linked to ongoing brain injury since its elevation has been observed in traumatic brain injury, post-cardiac arrest states, strokes, and dementia [38,45,107,110,111]. Similarly, KLK6 is seen in several neurodegeneration disorders [48,55,112,113,114,115]. Sustained elevations in TDP43, YKL40, and KLK6 may suggest potential links to delirium, increased psychoactive disorders, and atypical dementias in the wake of COVID-19. Variable increases in total and phosphorylated τ, and a predilection of individuals with pre-existing strokes to have another cerebrovascular event, further suggest the existence of vulnerable characteristics. NF-L was not dramatically altered, suggesting low dynamics of the neurodegeneration process, but a relatively low sensitivity test was used compared to the published data [50]. Using cerebrospinal fluid may have had a higher predictive value [57,116,117,118]. Elevations in serum NF-L levels were reported inconsistently in studies plagued with small fibers, unadjusted for comorbidities, or limited to the most sick patients [12,32,35,94,119,120,121]. Gisslen et al. reported the normalization of GFAP and NF-L six months after the resolution of COVID-19, but their patients continued to exhibit neurological symptoms and their study was limited to these markers [34]. Persistent elevations of several neurodegeneration markers suggest an ongoing pathological process in COVID survivors that is heterogeneous [38,43,44,46,51,52,55,87,94,111,122]. This indicates a potential mechanism for post-COVID-19 cognitive decline.

It is unclear if COVID-19 neurodegeneration is specific to SARS-CoV2 neurotoxicity, centrally mediated microglia inflammation, or the effect of ICU-grade inflammation [27,28,30,81,123]. Though SARS-CoV-2 particles can bind several proteins and affect their folding, several changes observed in our population occured after the resolution of acute inflammation [21,22,25]. A lack of correlation between viral protein load and increases in any of the neurodegeneration markers is consistent with observations linking the ongoing elevation of neurodegeneration markers in the cerebrospinal fluid without cytokine storms [120]. Alternatively, the smoldering of neuroinflammation may be the underlying cause of the persistent elevation of neurodegeneration markers, which is not severe enough to be reflected in the serum level of an acute neuro-injury [33,35]. The predominance of tissue inflammation in olfactory structures during COVID-19 suggests that generalized, or regional, inflammation is the culprit despite direct viral neurotoxicity [18,66,124]. Ferritin is the main marker of inflammation that correlated with TDP43 and YKL40 in our study. Furthermore, ferritin is used to gauge the degree of inflammation in several inflammatory processes and COVID-19 [122,125,126,127]. The activation of macrophages, frequently reflected by an increase in ferritin, was suggested as one of the major drivers of COVID-19 evolution and was related to the degree of neuronal degeneration and injury in general [26,83,122,125,128,129,130]. Elevations in CCL23, a cytokine involved in the recruitment of leukocytes to nervous system compartments and linked to vascular injury-related stroke, could suggest an influx of peripheral leukocytes into the brain [68,69,70]. Our study is correlational; however, several other investigations suggest that ongoing neuroinflammation leads to neurodegeneration [10,51,59,65,80,85]. Finally, the increase in neurodegeneration markers is part of the non-specific response to critical care illness [87]. This conclusion supports the finding that the cerebrospinal fluid of COVID-19 patients indicates a neuronal injury but not inflammation [120]. At the same time, the activation of leukocytes persists well into COVID-19 recovery, providing a pool of inflammatory cells [128]. These activated leukocytes can be recruited into the brain via a CCL23-driven mechanism [67,69,120]. Our data suggest that the activation of leukocytes continues despite a decline in S1, but the persistence of elevated procalcitonin and ferritin levels is highly suggestive of the smoldering of inflammation.

We demonstrated a sustained increase in NCAM-1, a marker traditionally linked to peripheral nervous system damage. NCAM was found to be 85% identical to SARS-CoV-2 envelope proteins in silica, suggesting that an immune response against SARS-CoV-2 could theoretically lead to demyelination and polyneuropathy via molecular mimicry [36]. Relatively high Guillain–Barre Syndrome (GBS) incidence among COVID-19 victims would further support this conclusion, but this is not conclusively documented in COVID-19 [3,29,37]. However, we found that neither the serum levels of spike protein nor the immunoglobulin titer against it correlated to the NCAM-1 increase. In addition, the level of inflammation measured via procalcitonin, ferritin, or IL-6 serum levels did not correlate with that of NCAM-1. NCAM-1 levels remained elevated during and after the onset of COVID-19, suggesting evidence of ongoing peripheral nerve injury. The next step is to link the severity of GBS to NCAM-1 levels to describe the correlation between these markers and the degree of clinical symptoms [131,132,133].

The persistent elevations of markers is accompanied by decreases in somre neuroprotective molecules during acute COVID-19 admission with some recovery afterwards [85,134,135]. Clusterin, TREM-2 and fetuin are linked to neuronal protection and cleavage of several pathological proteins [48,85,86,94,136,137]. The changes in their respective serum levels has been demonstrated in several inflammatory processes and is linked to the severity of the inflammatory response [97]. This acute TREM-2 initial decline would result in the increased vulnerability of COVID-19 survivors to secondary insults, further impairing their ability to successfully recover from neurological sequela. This raises concerns about the ability of survivors to recover fully from the disease if the initial insult deprived a host of some neuroprotective mechanisms [1,9,13,14,16]. An alteration in the spectrum of neurobiomarkers (protective vs. injury vs. degeneration) suggests a loss of autoregulated homeostasis and has been proposed to explain the origin of dementia [99,108]. Longitudinal surveillance, with a focus on determining a composite level of several biomarkers, may potentially identify survivors most vulnerable to the progress of clinically-evident dementia [33,56,70,86].

A significant finding of our work is that anti-viral treatment has a somewhat limited effect on the serum levels of neurodegeneration markers. Treatment with the convalescent plasma remdasavir seemed to limit the rise of some of the neurodological markers while steroids had no detectable statistically effect. This may suggest that the recovery of cognitive function may depend on limiting direct viral invasion as remdasavir is interferring with viral proliferation. [101,138]. Convalescent treatment may be particularly clinically applicable as it switches off activated T cells, an important culprit in COVID-19 neuropthology [139]. Neuroprotective markers may be the non-specific effect of suppressing inflammation as the leukocytes remain activated even after treatment [128,139].

Our data concur with prior observations regarding stroke being commonplace in patients with COVID-19 [7]. What is surprising is the late incidence of strokes. The primary determinant of stroke occurrence was the severity of the disease. Viral protein load or immunoglobulin levels play less significant roles compared to the overall severity of the disease. Furthermore, the activation of complement, vasculitis, and altered endothelium permeability is believed to be causative of the increased frequency of cerebrovascular accidents, delirium, and even psychiatric disorders in patients diagnosed with COVID-19 [13,20,67,74,140,141]. Furthermore, stroke progression in patients with pre-existing conditions depends on cytokines critical for arterial remodelings, like YKL-40 [141,142]. Finally, the increased incidence of strokes in COVID-19 patients may be mediated by other organ failures like cardiac irregularities, generalized coagulopathy, or general inflammation [140].

Our study has several limitations. The size of the group is relatively small but comparable to other studies [121]. Second, there may be an unintended bias in the recruitment of subjects considering the relatively high incidence of stroke as most of the patients admitted were critical during the initial part of the study. Over time, similar patients would not have qualified for hospital admission due to the paradigm shift of COVID-19 treatment favoring admission only for the most severe cases. This may be secondary to accidental enrollment bias as the recruitment area is located mostly within disadvantaged neighborhoods. However, our sample is very representative of the COVID-19 population. Neuro-ICU staff treated most COVID-19 patients, resulting in a higher awareness of neurological complications and subsequent cerebrovascular incidents. A total of 54 patients were lost at the 6-month follow-up, bringing the sample size at that time point to 51 patients. Consequently, the stroke incidence at 6 months represents the best-case scenario and is consistent with rates from prior reports [1,12,109]. Though we detected several changes in neuromarkers during COVID-19, the correction between them and the viral load was remote, while their correlations to inflammatory markers were much stronger. In any case, these are only correlational dependencies confounded by several factors like the time when the disease was clinically apparent, immunological make-up, and the genetic predisposition of the enrolled individuals. Elevations of markers are not highly specific for neurodegeneration as increases are also observed in several other conditions. We did not include a control group as the matching of patients is difficult and prone to bias. While most researchers focus on the viral load, we measured the viral burden by assessing the S-spike protein level. However, the pathological effect of SARS-CoV2 is related to both cytotoxic effect and immunological activation. RNA loads are more reflective of the former, while immunological response relates more to the latter. Using viral load is imprecise as several patients may acquire a disease with some lag before the emergence of clinically apparent symptoms. Furthermore, some of the markers are not specific to neurological injury or dementia [52,53,107,110,111,141]. YKL40 was linked to cardiovascular disorders, while TDB43 was elevated in several atypical dementias or ischemic damage to the brain in general [43,44,52,107,141]. Finally, we did not use the most sensitive tests to detect the levels of several neurodegeneration markers as we believed that COVID-19 inflammation would result in significant brain injury consistent with apparent increases in the serum levels of several markers [108]. We did not factor in genetic predisposition to several markers’ changes that significantly contribute to the susceptibility to neurodegeneration [98,108,143]. Finally, we were unable to assess several environmental and lifestyle factors, including diet, smoking, use of illicit drugs, pre-existing cognitive decline, and others. They have direct effects on the SARS-CoV-2 pathogen (initial viral load, immunosuppression status), modulate inflammation (Vit D3), or indirectly influence neurodegeneration processes (lipid profile) [144,145,146,147]. We could not adequately assess patients’ home intake as many of our patients reported not taking medications due to affordability. These are important factors as the neurodegenerative effect of COVID-19 inevitably affects patients with pre-existing conditions or recent critical care illnesses. The latter process may not have been resolved by the time of the COVID-19 exposure, resulting in compounding damage from two stressors.

Our study has several strengths. First, this is a longitudinal study of a significant cohort of patients adding to two prior studies [12,98]. Second, the longitudinal analysis of markers was shown to be more predictive of their recovery compared to singular measurements [121]. Third, we assessed several markers simultaneously in the context of immune system activation [7]. Some of the markers were assessed for the first time while others (YKL40, neurogranin) were reported in small groups [31]. Fourth, the study considered when the illness started, when the patients were admitted to the hospital, the severity of the clinical presentation, and the overall immune system activation. Finally, the study cohort is representative of the US population and diverse clinical presentations.

## 5. Conclusions

Our study revealed concomitant changes and changes in neurodegenerative and neuroprotective markers. We also showed changes in NCAM-1, a protein linked to the injury of peripheral nerves. The observed patterns of biomarker changes had traits typical of a post-ICU syndrome.

## Figures and Tables

**Table 1:** Demographical and clinical variables of the studied population at the beginning of data collection compared to patients at the 6-month follow-up, patients who had a stroke six months later, and patients 48 h after admission.

		All Patients Recruited vs. Patients Available to Follow-Up at 6 Months.		Comparison of All Patients Recruited Who Experienced a Cerebrovascular Event (CVA) by the 6 Month Follow-Up vs. Patients with No Post-COVID-19 CVA.			
		**All (n = 105)**	**6 months (n = 51)**	**No Stroke (n = 95)**		**Stroke (n = 10)**	
	Age [X ± SD]		62.4 ± 15.52	58.4 + 18.50		64.1 ± 15.07	
Age	Below 60 [%]	43.4	33.9	44.2		40.0	
	Over 60 [%]	56.6	66.1	55.8		60.0	
Gender	Male [%]	60.0	62.7	57.9		80.0	
	Female [%]	40.0	37.3	42.1		20.0	
Height	Meters [X ± SD]	1.71 ± 0.08	1.72 ± 0.10	1.70 ± 0.10 *		1.74 ± 0.12 *	
Weight	Kilograms [X ± SD]	93.1 ± 20.11	88.9 ± 24.44	93.4 ± 27.72		90.6 ± 21.85	
Race	Hispanic Latino [%]	27.6	8.5	26.3		40.0	
	Black [%]	62.9	61.0	64.2		50.0	
	Other/UNK/Asian [%]	9.5	30.5	9.5		10.0	
Clinical characteristics		**All (n = 105)**	**6 months (n = 51)**	**No Stroke (n = 95)**		**Stroke (n = 10)**	
Mortality [%]		21.9	32.2	20.0		40.0	
Length of Stay [days; X ± SD]		**16.6 ± 14.18 ^#^**	**38.0 ± 31.21 ^#^**	**15.5 ± 22.88**		**17.4 ± 24.21**	
ICU [%]		50.0	71.2	49.5		63.6	
Intubated [%]		33.0	50.8	33.7		30.0	
ECMO [%]		9.4	15.3	7.4		30.0	
APACHE + 1 h [X ± SD]		11.0 ± 6.26	12.6 ± 7.88	10.8 ± 8.01		14.1 ± 4.79	
APACHE + 24 h [X ± SD]		11.0 ± 5.83	12.9 ± 7.05	10.5 ± 7.45 *		16.1 ± 2.69 *	
MOF		**All (n = 105)**		**No Stroke (n = 95)**		**Stroke (n = 10)**	
		Admission	48 h	Admission	48 h	Admission	48 h
MODS [X ± SD]		3.0 ± 2.72	3.2 ± 2.96	2.9 ± 2.71	3.3 ± 3.06	4.7 ± 2.31 **	2.3 ± 1.64 **

* Statistical difference when comparing patients with stroke vs. patients without stroke. ** Statistical difference when comparing patients at admission vs. patients at 48 h from admission. # Statistical difference when comparing patients at admission vs. patients at 6 months from admission.

**Table 2:** Dynamics of inflammatory markers over time in all patients, and those who did vs. did not suffer from a stroke within 6 months following hospitalization for COVID-19.

		Admission—48 h	2d–4d	5d–7d	8–25 Days
**CCL23**	All	434.4 ± 578.05	594.1 ± 717.13	886 ± 1001.68	1069.1 = ±1165.81 *
	Stroke	880.1 ± 1232.56	466.5 ± 696.57	644 ± 1113.75	1640.4 ± 2198.53
	No Stroke	413.2 ± 874.78	610.4 ± 1169.72	917.6 ± 1348.26	905.8 ± 1302.94
		**Admission—48 h**	**2d–4d**	**5d–7d**	**8–25 days**
MCP-1	All	449.4 ± 499.09	458.4 ± 743.52	291.4 ± 304.24 *	601.8 ± 619.20
	Stroke	374.6 ± 0.0	420.4 ± 415.73	145.1 ± 126.60	324.2 ± 334.15
	No Stroke	451.4 ± 505.82	463.7 ± 781.66	317.2 ± 321.24	681.1 ± 663.93
		**Admission—48 h**	**2d–4d**	**5d–7d**	**8–25 days**
MIF	All	258.4 ± 313.07	187.6 ± 186.1 *	182.3 ± 124.89 *	372.9 ± 1101.91
	Stroke	214.6 ± 173.22	169.32 ± 135.59	152.6 ± 93.10	270.3699 ± 399.22
	No Stroke	260.5 ± 322.97	**190.1 ± 194.87 ^#^**	**185.8 ± 129.10 ^#^**	399.4 ± 12223.63
		**Admission—48 h**	**2d–4d**	**5d–7d**	**7–28 days**
IL-6	All	10.9 ± 11.98	13.1 ± 13.81	11.9 ± 11.98	11.2 ± 10.27
	Stroke	2.8 ± 0.15	10.4 ± 23.07	**21.6 ± 35.56 ^&^**	**23.6 ± 32.61 ^&^**
	No Stroke	11.4 ± 16.88	13.5 ± 21.12	**10.9 ± 14.95 ^&^**	**8.4 ± 11.43 ^&^**
		**Admission—48 h**	**2d–4d**	**5d–7d**	**8–25 days**
IL-8	All	22.2 ± 30.7	10.4 ± 10.36 *	16.9 ± 12.60	11 ± 6.82 *
	Stroke	12.7 ± N/A	**5.2 ± 2.70 ^##^**	2.6 ± N/A	8.2 ± 5.10
	No Stroke	22.5 ± 57.15	**10.8 ± 19.89 ^#^**	17.9 ± 20.96	**11.7 ± 10.36 ^#^**
		**Admission—48 h**	**2d–4d**	**5d–7d**	**8–25 days**
TNFα	All	0.6 ± 0.42	1.1 ± 0.98 *	1.2 ± 1.07	0.7 ± 0.40
	Stroke	0.5 ± 0.55	1.7 ± 2.36	0.7 ± 0.23	0.9 ± 0.92
	No Stroke	0.6 ± 0.54	1 ± 1.69	1.2 ± 2.74	0.7 ± 0.52
		**Admission—48 h**	**2d–4d**	**5d–7d**	**8–25 days**
Platelet Count	All	218.7 ± 68.17	252.8 ± 93.7 *	280.8 ± 108.02 *	239.2 ± 78.71
	Stroke	232.8 ± 81.61	264.4 ± 122.43	321.4 ± 119.03	254.5 ± 137.97
	No Stroke	217.1 ± 87.37	**251.5 ± 125 ^#^**	**277.4 ± 146.61 ^#^**	237.8 ± 98.69
		**Admission—48 h**	**2d–4d**	**5d–7d**	**8–25 days**
WBC Count	All	8.2 ± 3.41	9 ± 3.65	11 ± 4.23 *	11.4 ± 4.95 *
	Stroke	7.1 ± 2.99	7.1 ± 3.09	11.9 ± 4.3	**17.6 ± 17.07 ^&^**
	No Stroke	8.3 ± 4.39	9.2 ± 4.78	**10.9 ± 6.99 ^#^**	**10.8 ± 5.73 ^#,&^**

Treat. * Statistical difference when compared to all patients at admission (*p* < 0.046). ^#^ Statistical difference when compared to patients who did not have a stroke at admission (*p* < 0.012). ^##^ Statistical difference when compared to patients who did have a stroke at admission (*p* < 0.014). ^&^ Statistical difference when comparing patients who did vs. did not have a stroke at a given timepoint (*p* < 0.05).

## Data Availability

The datasets used and/or analyzed during the current study are available from the corresponding authors on reasonable request.

## Supplementary Materials

The following are available online at https://www.mdpi.com/article/10.3390/biomedicines9121791/s1. Figure S1: The distribution of serum samples from admission; Figure S2: Patient disposition at one- and six- months post-hospital admission; Figure S3: Serum levels off phosphorylated tau (A) and total tau (B) were highly variable; Figure S4: Correlations between viral burden, COVID-19 direct immune system response, and neurodegeneration markers at admission (A) and throughout patient stay (B); Table S1: Comparison of markers between all patients, and patients with vs without stroke.

## References

[1] Kumar S., Veldhuis A., Malhotra T. (2021). Neuropsychiatric and Cognitive Sequelae of COVID-19. Front. Psychol..

[2] Li Z., Li X., Shen J., Chan M.T.V., Wu W.K.K. (2021). Miller Fisher syndrome associated with COVID-19, an up-to-date systematic review. Environ. Sci. Pollut. Res. Int..

[3] Stoian A., Bălașa R., Grigorescu B.L., Maier S., Andone S., Cocuz I.G., Bajko Z., Filep C.R., Stoian M. (2021). Guillain-Barré syndrome associated with Covid-19, A close relationship or just a coincidence? (Review). Exp. Med..

[4] Jung M., Rujescu D. (2020). Immune cell puzzle COVID-19, how do SARS-CoV infections contribute to psychiatric diseases?. Eur. Arch. Psychiatry Clin. Neurosci..

[5] Romagnolo A., Imbalzano G., Artusi C.A., Balestrino R., Ledda C., De Rosa F.G., Riccardini F., Montanaro E., Bozzali M., Rizzone M.G. (2021). Neurological comorbidities and COVID-19-related case fatality: A cohort study. J. Neurol. Sci..

[6] Espíndola O.M., Brandão C.O., Gomes Y.C.P., Siqueira M., Soares C.N., Lima M., Leite A., Torezani G., Araujo A.Q.C., Silva M.T.T. (2021). Cerebrospinal fluid findings in neurological diseases associated with COVID-19 and insights into mechanisms of disease development. Int. J. Infect. Dis..

[7] Lambrecq V., Hanin A., Munoz-Musat E., Chougar L., Gassama S., Delorme C., Cousyn L., Borden A., Damiano M., Frazzini V. (2021). Association of Clinical, Biological, and Brain Magnetic Resonance Imaging Findings With Electroencephalographic Findings for Patients With COVID-19. JAMA Netw. Open.

[8] LaHue S.C., Douglas V.C., Miller B.L. (2020). The One-Two Punch of Delirium and Dementia During the COVID-19 Pandemic and Beyond. Front. Neurol..

[9] Delorme C., Houot M., Rosso C., Carvalho S., Nedelec T., Maatoug R., Pitron V., Gassama S., Sambin S., Bombois S. (2021). The wide spectrum of COVID-19 neuropsychiatric complications within a multidisciplinary centre. Brain Commun..

[10] Damiano R.F., Guedes B.F., de Rocca C.C., de Pádua Serafim A., Castro L.H.M., Munhoz C.D., Nitrini R., Filho G.B., Miguel E.C., Lucchetti G. (2021). Cognitive decline following acute viral infections: Literature review and projections for post-COVID-19. Eur. Arch. Psychiatry Clin. Neurosci..

[11] McLoughlin B.C., Miles A., Webb T.E., Knopp P., Eyres C., Fabbri A., Humphries F., Davis D. (2020). Functional and cognitive outcomes after COVID-19 delirium. Eur. Geriatr. Med..

[12] Sun B., Tang N., Peluso M.J., Iyer N.S., Torres L., Donatelli J.L., Munter S.E., Nixon C.C., Rutishauser R.L., Rodriguez-Barraquer I. (2021). Characterization and Biomarker Analyses of Post-COVID-19 Complications and Neurological Manifestations. Cells.

[13] Yong E. (2020). Long-Haulers Are Redefining COVID-19. Atlantic.

[14] Carbajal E. (2021). CDC Releases Framework for Treating COVID-19 Long-Haulers, Becker’s Hospitla Review.

[15] Ibanez A., Santamaria-Garcia H., Guerrero Barragan A., Kornhuber A., Ton A.M.M., Slachevsky A., Teixeira A.L., Mar Meza B.M., Serrano C.M., Cano C. (2020). The impact of SARS-CoV-2 in dementia across Latin America: A call for an urgent regional plan and coordinated response. Alzheimers Dement..

[16] Meier I.B., Vieira Ligo Teixeira C., Tarnanas I., Mirza F., Rajendran L. (2021). Neurological and mental health consequences of COVID-19, potential implications for well-being and labour force. Brain Commun..

[17] Crunfli F., Carregari V.C., Veras F.P., Vendramini P.H., Valença A.G.F., Marcelo Antunes A.S.L., Brandão-Teles C., Zuccoli G.d.S., Reis-de-Oliveira G., Silva-Costa L.C. (2021). SARS-CoV-2 infects brain astrocytes of COVID-19 patients and impairs neuronal viability. medRxiv.

[18] Klingenstein M., Klingenstein S., Neckel P.H., Mack A.F., Wagner A.P., Kleger A., Liebau S., Milazzo A. (2020). Evidence of SARS-CoV2 Entry Protein ACE2 in the Human Nose and Olfactory Bulb. Cells Tissues Organs.

[19] Netland J., Meyerholz D.K., Moore S., Cassell M., Perlman S. (2008). Severe acute respiratory syndrome coronavirus infection causes neuronal death in the absence of encephalitis in mice transgenic for human ACE2. J. Virol..

[20] Virhammar J., Kumlien E., Fällmar D., Frithiof R., Jackmann S., Sköld M.K., Kadir M., Frick J., Lindeberg J., Olivero-Reinius H. (2020). Acute necrotizing encephalopathy with SARS-CoV-2 RNA confirmed in cerebrospinal fluid. Neurology.

[21] Ramani A., Müller L., Ostermann P.N., Gabriel E., Abida-Islam P., Müller-Schiffmann A., Mariappan A., Goureau O., Gruell H., Walker A. (2020). SARS-CoV-2 targets neurons of 3D human brain organoids. EMBO J..

[22] Idrees D., Kumar V. (2021). SARS-CoV-2 spike protein interactions with amyloidogenic proteins: Potential clues to neurodegeneration. Biochem. Biophys. Res. Commun..

[23] Hsu J.T., Tien C.F., Yu G.Y., Shen S., Lee Y.H., Hsu P.C., Wang Y., Chao P.K., Tsay H.J., Shie F.S. (2021). The Effects of Aβ(1-42) Binding to the SARS-CoV-2 Spike Protein S1 Subunit and Angiotensin-Converting Enzyme 2. Int. J. Mol. Sci..

[24] Cascarina S.M., Ross E.D. (2020). A proposed role for the SARS-CoV-2 nucleocapsid protein in the formation and regulation of biomolecular condensates. Faseb J..

[25] Gordon D.E., Hiatt J., Bouhaddou M., Rezelj V.V., Ulferts S., Braberg H., Jureka A.S., Obernier K., Guo J.Z., Batra J. (2020). Comparative host-coronavirus protein interaction networks reveal pan-viral disease mechanisms. Science.

[26] DiMeglio M., Furey W., Hajj J., Lindekens J., Patel S., Acker M., Bavaria J., Szeto W.Y., Atluri P., Haber M. (2019). Observational study of long-term persistent elevation of neurodegeneration markers after cardiac surgery. Sci. Rep..

[27] Teeters D.A., Moua T., Li G., Kashyap R., Biehl M., Kaur R., Gajic O., Boeve B.F., St Louis E.K., Petersen R.C. (2016). Mild Cognitive Impairment and Risk of Critical Illness. Crit Care Med..

[28] Ramalho J., Castillo M. (2015). Dementia resulting from traumatic brain injury. Dement. NeuroPsychol..

[29] Hasliza A.H., Tohid H., Loh K.Y., Santhi P. (2015). Post dengue neurological complication. Malays. Fam Physician.

[30] Yiannopoulou K.G., Anastasiou I.P., Ganetsos T.K., Efthimiopoulos P., Papageorgiou S.G. (2012). Prevalence of dementia in elderly patients with hip fracture. Hip. Int..

[31] Pilotto A., Masciocchi S., Volonghi I., De Giuli V., Caprioli F., Mariotto S., Ferrari S., Bozzetti S., Imarisio A., Risi B. (2021). SARS-CoV-2 encephalitis is a cytokine release syndrome: Evidences from cerebrospinal fluid analyses. Clin. Infect. Dis..

[32] Virhammar J., Nääs A., Fällmar D., Cunningham J.L., Klang A., Ashton N.J., Jackmann S., Westman G., Frithiof R., Blennow K. (2020). Biomarkers for central nervous system injury in cerebrospinal fluid are elevated in COVID-19 and associated with neurological symptoms and disease severity. Eur. J. Neurol..

[33] Ameres M., Brandstetter S., Toncheva A.A., Kabesch M., Leppert D., Kuhle J., Wellmann S. (2020). Association of neuronal injury blood marker neurofilament light chain with mild-to-moderate COVID-19. J. Neurol..

[34] Kanberg N., Simrén J., Edén A., Andersson L.M., Nilsson S., Ashton N.J., Sundvall P.D., Nellgård B., Blennow K., Zetterberg H. (2021). Neurochemical signs of astrocytic and neuronal injury in acute COVID-19 normalizes during long-term follow-up. EBioMedicine.

[35] De Lorenzo R., Loré N.I., Finardi A., Mandelli A., Cirillo D.M., Tresoldi C., Benedetti F., Ciceri F., Rovere-Querini P., Comi G. (2021). Blood neurofilament light chain and total tau levels at admission predict death in COVID-19 patients. J. Neurol..

[36] Morsy S. (2020). NCAM protein and SARS-COV-2 surface proteins: In-silico hypothetical evidence for the immunopathogenesis of Guillain-Barré syndrome. Med. Hypotheses.

[37] Leis A.A., Stokic D.S. (2016). Zika Virus and Guillain-Barre Syndrome: Is There Sufficient Evidence for Causality?. Front. Neurol..

[38] Umahara T., Uchihara T., Hirao K., Shimizu S., Hanyu H. (2020). Phosphorylated TDP-43 localizes to chronic cerebral infarctions in human brains. Histol. Histopathol..

[39] Pereira J.B., Janelidze S., Ossenkoppele R., Kvartsberg H., Brinkmalm A., Mattsson-Carlgren N., Stomrud E., Smith R., Zetterberg H., Blennow K. (2021). Untangling the association of amyloid-β and tau with synaptic and axonal loss in Alzheimer’s disease. Brain.

[40] Blennow K. (2017). A Review of Fluid Biomarkers for Alzheimer’s Disease: Moving from CSF to Blood. Neurol. Ther..

[41] Mattsson N., Insel P.S., Palmqvist S., Portelius E., Zetterberg H., Weiner M., Blennow K., Hansson O. (2016). Alzheimer’s Disease Neuroimaging I: Cerebrospinal fluid tau, neurogranin, and neurofilament light in Alzheimer’s disease. EMBO Mol. Med..

[42] Cicognola C., Janelidze S., Hertze J., Zetterberg H., Blennow K., Mattsson-Carlgren N., Hansson O. (2021). Plasma glial fibrillary acidic protein detects Alzheimer pathology and predicts future conversion to Alzheimer dementia in patients with mild cognitive impairment. Alzheimers Res..

[43] Hellwig K., Kvartsberg H., Portelius E., Andreasson U., Oberstein T.J., Lewczuk P., Blennow K., Kornhuber J., Maler J.M., Zetterberg H. (2015). Neurogranin and YKL-40, independent markers of synaptic degeneration and neuroinflammation in Alzheimer’s disease. Alzheimers Res..

[44] Craig-Schapiro R., Perrin R.J., Roe C.M., Xiong C., Carter D., Cairns N.J., Mintun M.A., Peskind E.R., Li G., Galasko D.R. (2010). YKL-40, a novel prognostic fluid biomarker for preclinical Alzheimer’s disease. Biol. Psychiatry.

[45] James B.D., Wilson R.S., Boyle P.A., Trojanowski J.Q., Bennett D.A., Schneider J.A. (2016). TDP-43 stage, mixed pathologies, and clinical Alzheimer’s-type dementia. Brain.

[46] De Boer E.M.J., Orie V.K., Williams T., Baker M.R., De Oliveira H.M., Polvikoski T., Silsby M., Menon P., van den Bos M., Halliday G.M. (2021). TDP-43 proteinopathies: A new wave of neurodegenerative diseases. J. Neurol. Neurosurg. Psychiatry.

[47] Patra K., Soosaipillai A., Sando S.B., Lauridsen C., Berge G., Møller I., Grøntvedt G.R., Bråthen G., Begcevic I., Moussaud S. (2018). Assessment of kallikrein 6 as a cross-sectional and longitudinal biomarker for Alzheimer’s disease. Alzheimers Res..

[48] Dukic L., Simundic A.M., Martinic-Popovic I., Kackov S., Diamandis A., Begcevic I., Diamandis E.P. (2016). The role of human kallikrein 6, clusterin and adiponectin as potential blood biomarkers of dementia. Clin. Biochem..

[49] Wilson R.S., Yu L., Trojanowski J.Q., Chen E.-Y., Boyle P.A., Bennett D.A., Schneider J.A. (2013). TDP-43 pathology, cognitive decline, and dementia in old age. JAMA Neurol..

[50] Mattsson N., Andreasson U., Zetterberg H., Blennow K. (2017). Association of Plasma Neurofilament Light With Neurodegeneration in Patients With Alzheimer Disease. JAMA Neurol..

[51] Thammisetty S.S., Pedragosa J., Weng Y.C., Calon F., Planas A., Kriz J. (2018). Age-related deregulation of TDP-43 after stroke enhances NF-κB-mediated inflammation and neuronal damage. J. Neuroinflamm..

[52] Lee E.B., Lee V.M.Y., Trojanowski J.Q., Neumann M. (2008). TDP-43 immunoreactivity in anoxic, ischemic and neoplastic lesions of the central nervous system. Acta Neuropathol..

[53] Scarisbrick I.A., Yoon H., Panos M., Larson N., Blaber S.I., Blaber M., Rodriguez M. (2012). Kallikrein 6 regulates early CNS demyelination in a viral model of multiple sclerosis. Brain Pathol..

[54] Martínez-Morillo E., Diamandis A., Romaschin A.D., Diamandis E.P. (2012). Kallikrein 6 as a serum prognostic marker in patients with aneurysmal subarachnoid hemorrhage. PLoS ONE.

[55] Bando Y., Hagiwara Y., Suzuki Y., Yoshida K., Aburakawa Y., Kimura T., Murakami C., Ono M., Tanaka T., Jiang Y.P. (2018). Kallikrein 6 secreted by oligodendrocytes regulates the progression of experimental autoimmune encephalomyelitis. Glia.

[56] Sutphen C.L., Jasielec M.S., Shah A.R., Macy E.M., Xiong C., Vlassenko A.G., Benzinger T.L., Stoops E.E., Vanderstichele H.M., Brix B. (2015). Longitudinal Cerebrospinal Fluid Biomarker Changes in Preclinical Alzheimer Disease During Middle Age. JAMA Neurol..

[57] Mattsson N., Cullen N.C., Andreasson U., Zetterberg H., Blennow K. (2019). Association Between Longitudinal Plasma Neurofilament Light and Neurodegeneration in Patients With Alzheimer Disease. JAMA Neurol..

[58] Ryabkova V.A., Churilov L.P., Shoenfeld Y. (2021). Influenza infection, SARS, MERS and COVID-19, Cytokine storm—The common denominator and the lessons to be learned. Clin. Immunol..

[59] Minagar A., Shapshak P., Fujimura R., Ownby R., Heyes M., Eisdorfer C. (2002). The role of macrophage/microglia and astrocytes in the pathogenesis of three neurologic disorders: HIV-associated dementia, Alzheimer disease, and multiple sclerosis. J. Neurol. Sci..

[60] Xiang Z., Haroutunian V., Ho L., Purohit D., Pasinetti G.M. (2006). Microglia activation in the brain as inflammatory biomarker of Alzheimer’s disease neuropathology and clinical dementia. Dis. Markers.

[61] Garwood C.J., Ratcliffe L.E., Simpson J.E., Heath P.R., Ince P.G., Wharton S.B. (2017). Review: Astrocytes in Alzheimer’s disease and other age-associated dementias: A supporting player with a central role. Neuropathol. Appl. Neurobiol..

[62] Zhan X., Stamova B., Sharp F.R. (2018). Lipopolysaccharide Associates with Amyloid Plaques, Neurons and Oligodendrocytes in Alzheimer’s Disease Brain: A Review. Front. Aging Neurosci..

[63] Mackenzie I.R.A. (2000). Activated microglia in dementia with Lewy bodies. Neurology.

[64] Keren-Shaul H., Spinrad A., Weiner A., Matcovitch-Natan O., Dvir-Szternfeld R., Ulland T.K., David E., Baruch K., Lara-Astaiso D., Toth B. (2017). A Unique Microglia Type Associated with Restricting Development of Alzheimer’s Disease. Cell.

[65] Torres K.C., Araujo Pereira P., Lima G.S., Bozzi I.C., Rezende V.B., Bicalho M.A., Moraes E.N., Miranda D.M., Romano-Silva M.A. (2013). Increased frequency of T cells expressing IL-10 in Alzheimer disease but not in late-onset depression patients. Prog. Neuropsychopharmacol. Biol. Psychiatry.

[66] Takeda S., Sato N., Morishita R. (2014). Systemic inflammation, blood-brain barrier vulnerability and cognitive/non-cognitive symptoms in Alzheimer disease: Relevance to pathogenesis and therapy. Front. Aging Neurosci..

[67] Vaschetto R., Cena T., Sainaghi P.P., Meneghetti G., Bazzano S., Vecchio D., Pirisi M., Brustia D., Barini M., Cammarota G. (2020). Cerebral nervous system vasculitis in a COVID-19 patient with pneumonia. J. Clin. Neurosci..

[68] Wang X., Yang Y., Zhao Z., Li P., Ma C., Zhu B. (2020). Diagnostic Value of Serum MIF and CCL23 in the Patients with Acute Cerebral Infarction. Clin. Lab..

[69] Simats A., García-Berrocoso T., Penalba A., Giralt D., Llovera G., Jiang Y., Ramiro L., Bustamante A., Martinez-Saez E., Canals F. (2018). CCL23, a new CC chemokine involved in human brain damage. J. Intern. Med..

[70] Faura J., Bustamante A., Penalba A., Giralt D., Simats A., Martínez-Sáez E., Alcolea D., Fortea J., Lleó A., Teunissen C.E. (2020). CCL23, A Chemokine Associated with Progression from Mild Cognitive Impairment to Alzheimer’s Disease. J. Alzheimers Dis..

[71] Bielekova B., Komori M., Xu Q., Reich D.S., Wu T. (2012). Cerebrospinal fluid IL-12p40, CXCL13 and IL-8 as a combinatorial biomarker of active intrathecal inflammation. PLoS ONE.

[72] Sokolova A., Hill M.D., Rahimi F., Warden L.A., Halliday G.M., Shepherd C.E. (2009). Monocyte Chemoattractant Protein-1 Plays a Dominant Role in the Chronic Inflammation Observed in Alzheimer’s Disease. Brain Pathol..

[73] Xu Y., Shen Y.-Y., Zhang X.-P., Gui L., Cai M., Peng G.-P., Pan X.-D., Zhang J., Gan D., Li B. (2020). Diagnostic potential of urinary monocyte chemoattractant protein-1 for Alzheimer’s disease and amnestic mild cognitive impairment. Eur. J. Neurol..

[74] Uemura M.T., Maki T., Ihara M., Lee V.M.Y., Trojanowski J.Q. (2020). Brain Microvascular Pericytes in Vascular Cognitive Impairment and Dementia. Front. Aging Neurosci..

[75] Sprung J., Warner D.O., Knopman D.S., Petersen R.C., Mielke M.M., Jack C.R., Jr Martin D.P., Hanson A.C., Schroeder D.R., Przybelski S.A. (2021). Brain MRI after critical care admission: A longitudinal imaging study. J. Crit Care.

[76] Lu Y., Li X., Geng D., Mei N., Wu P.Y., Huang C.C., Jia T., Zhao Y., Wang D., Xiao A. (2020). Cerebral Micro-Structural Changes in COVID-19 Patients—An MRI-based 3-month Follow-up Study. EClinicalMedicine.

[77] Kandemirli S.G., Dogan L., Sarikaya Z.T., Kara S., Akinci C., Kaya D., Kaya Y., Yildirim D., Tuzuner F., Yildirim M.S. (2020). Brain MRI Findings in Patients in the Intensive Care Unit with COVID-19 Infection. Radiology.

[78] Zhou H., Lu S., Chen J., Wei N., Wang D., Lyu H., Shi C., Hu S. (2020). The landscape of cognitive function in recovered COVID-19 patients. J. Psychiatr. Res..

[79] Almeria M., Cejudo J.C., Sotoca J., Deus J., Krupinski J. (2020). Cognitive profile following COVID-19 infection: Clinical predictors leading to neuropsychological impairment. Brain Behav. Immun. Health.

[80] Chen Y., Wang J., Liu C., Su L., Zhang D., Fan J., Yang Y., Xiao M., Xie J., Xu Y. (2020). IP-10 and MCP-1 as biomarkers associated with disease severity of COVID-19. Mol. Med..

[81] Laudanski K., Jihane H., Antalosky B., Ghani D., Phan U., Hernandez R., Okeke T., Wu J., Rader D., Susztak K. (2021). Unbiased Analysis of Temporal Changes in Immune Serum Markers in Acute COVID-19 Infection With Emphasis on Organ Failure, Anti-Viral Treatment, and Demographic Characteristics. Front. Immunol..

[82] Ray R., Juranek J.K., Rai V. (2016). RAGE axis in neuroinflammation, neurodegeneration and its emerging role in the pathogenesis of amyotrophic lateral sclerosis. Neurosci. Biobehav. Rev..

[83] Collins-Praino L.E., Corrigan F. (2017). Does neuroinflammation drive the relationship between tau hyperphosphorylation and dementia development following traumatic brain injury?. Brain Behav. Immun..

[84] Sita G., Graziosi A., Hrelia P., Morroni F. (2021). NLRP3 and Infections: β-Amyloid in Inflammasome beyond Neurodegeneration. Int. J. Mol. Sci..

[85] Monllor P., Giraldo E., Badia M.C., de la Asuncion J.G., Alonso M.D., Lloret A., Vina J. (2021). Serum Levels of Clusterin, PKR, and RAGE Correlate with Amyloid Burden in Alzheimer’s Disease. J. Alzheimers Dis..

[86] Smith E.R., Nilforooshan R., Weaving G., Tabet N. (2011). Plasma fetuin-A is associated with the severity of cognitive impairment in mild-to-moderate Alzheimer’s disease. J. Alzheimers Dis..

[87] Poloni T.E., Medici V., Moretti M., Visonà S.D., Cirrincione A., Carlos A.F., Davin A., Gagliardi S., Pansarasa O., Cereda C. (2021). COVID-19-related neuropathology and microglial activation in elderly with and without dementia. Brain Pathol..

[88] Perunicic-Pekovic G., Pljesa S., Rasic-Milutinovic Z., Stankovic S., Ilic M., Maletic R. (2008). Inflammatory cytokines and malnutrition as related to risk for cardiovascular disease in hemodialysis patients. Can. J. Physiol. Pharm..

[89] Izquierdo Delgado E., Gutiérrez Ríos R., Andrés Calvo M., Repiso Gento I., Castrillo Sanz A., Rodríguez Herrero R., Rodríguez Sanz M.F., Tola-Arribas M.A. (2021). Nutritional status assessment in Alzheimer disease and its influence on disease progression. Neurologia.

[90] Koh S.S., Ooi S.C., Lui N.M., Qiong C., Ho L.T., Cheah I.K., Halliwell B., Herr D.R., Ong W.Y. (2021). Effect of Ergothioneine on 7-Ketocholesterol-Induced Endothelial Injury. Neuromol. Med..

[91] Jirak P., Stechemesser L., Moré E., Franzen M., Topf A., Mirna M., Paar V., Pistulli R., Kretzschmar D., Wernly B., Makowski G.S. (2019). Chapter Three—Clinical Implications of Fetuin-A, Advances in Clinical Chemistry.

[92] Gratuze M., Leyns C.E.G., Holtzman D.M. (2018). New insights into the role of TREM2 in Alzheimer’s disease. Mol. Neurodegener..

[93] Raha A.A., Henderson J.W., Stott S.R., Vuono R., Foscarin S., Friedland R.P., Zaman S.H., Raha-Chowdhury R. (2017). Neuroprotective Effect of TREM-2 in Aging and Alzheimer’s Disease Model. J. Alzheimers Dis..

[94] Romero J.R., Demissie S., Beiser A., Himali J.J., DeCarli C., Levy D., Seshadri S. (2020). Relation of plasma β-amyloid, clusterin, and tau with cerebral microbleeds: Framingham Heart Study. Ann. Clin. Transl. Neurol..

[95] Rodríguez-Rivera C., Garcia M.M., Molina-Álvarez M., González-Martín C., Goicoechea C. (2021). Clusterin: Always protecting. Synthesis, function and potential issues. Biomed. Pharmacother..

[96] Falgarone G., Chiocchia G. (2009). Chapter 8 Clusterin: A Multifacet Protein at the Crossroad of Inflammation and Autoimmunity, Advances in Cancer Research.

[97] Hassan A.M., Saleh E.S., Strak S.K. (2014). Evaluation of Fetuin-A Protein and Some Inflammatory Biomarkers in Patients with Coronary Artery Disease. Am. J. Pharmacol. Sci..

[98] Xiong N., Schiller M.R., Li J., Chen X., Lin Z. (2021). Severe COVID-19 in Alzheimer’s disease: APOE4’s fault again?. Alzheimers Res..

[99] Khullar S., Wang D. (2021). Integrative analysis of multi-omics reveals gene regulatory networks across brain regions from risk variants to phenotypes of Alzheimer’s disease and Covid-19. bioRxiv.

[100] Roback J.D., Guarner J. (2020). Convalescent Plasma to Treat COVID-19, Possibilities and Challenges. JAMA.

[101] Rezagholizadeh A., Khiali S., Sarbakhsh P., Entezari-Maleki T. (2021). Remdesivir for treatment of COVID-19; an updated systematic review and meta-analysis. Eur. J. Pharm..

[102] Wu C., Hou D., Du C., Cai Y., Zheng J., Xu J., Chen X., Chen C., Hu X., Zhang Y. (2020). Corticosteroid therapy for coronavirus disease 2019-related acute respiratory distress syndrome: A cohort study with propensity score analysis. Crit Care.

[103] Knaus W.A., Draper E.A., Wagner D.P., Zimmerman J.E. (1985). APACHE II: A severity of disease classification system. Crit Care Med..

[104] Bian Y., Zhang P., Xiong Y., Xu F., Zhu S., Tang Z., Xue Z. (2015). Application of the APACHE II score to assess the condition of patients with critical neurological diseases. Acta Neurol. Belg..

[105] Cleves M.A., Sanchez N., Draheim M. (1997). Evaluation of two competing methods for calculating Charlson’s comorbidity index when analyzing short-term mortality using administrative data. J. Clin. Epidemiol..

[106] Peres Bota D., Melot C., Lopes Ferreira F., Nguyen Ba V., Vincent J.L. (2002). The Multiple Organ Dysfunction Score (MODS) versus the Sequential Organ Failure Assessment (SOFA) score in outcome prediction. Intensive Care Med..

[107] Davidson Y.S., Raby S., Foulds P.G., Robinson A., Thompson J.C., Sikkink S., Yusuf I., Amin H., DuPlessis D., Troakes C. (2011). TDP-43 pathological changes in early onset familial and sporadic Alzheimer’s disease, late onset Alzheimer’s disease and Down’s syndrome: Association with age, hippocampal sclerosis and clinical phenotype. Acta Neuropathol..

[108] Duits F.H., Wesenhagen K.E.J., Ekblad L., Wolters E., Willemse E.A.J., Scheltens P., van der Flier W.M., Teunissen C.E., Visser P.J., Tijms B.M. (2021). Four subgroups based on tau levels in Alzheimer’s disease observed in two independent cohorts. Alzheimers Res..

[109] Yelin D., Wirtheim E., Vetter P., Kalil A.C., Bruchfeld J., Runold M., Guaraldi G., Mussini C., Gudiol C., Pujol M. (2020). Long-term consequences of COVID-19, research needs. Lancet Infect. Dis..

[110] He T., Zuo Y., Ai-Zakwani K., Luo J., Zhu H., Yan X.X., Liu F. (2018). Subarachnoid hemorrhage enhances the expression of TDP-43 in the brain of experimental rats and human subjects. Exp. Med..

[111] Hatsuta H., Takao M., Nogami A., Uchino A., Sumikura H., Takata T., Morimoto S., Kanemaru K., Adachi T., Arai T. (2019). Tau and TDP-43 accumulation of the basal nucleus of Meynert in individuals with cerebral lobar infarcts or hemorrhage. Acta Neuropathol. Commun..

[112] Sharma A., Chunduri A., Gopu A., Shatrowsky C., Crusio W.E., Delprato A. (2020). Common genetic signatures of Alzheimer’s disease in Down Syndrome. F1000Res.

[113] Mella C., Figueroa C.D., Otth C., Ehrenfeld P. (2020). Involvement of Kallikrein-Related Peptidases in Nervous System Disorders. Front. Cell Neurosci..

[114] Drucker K.L., Gianinni C., Decker P.A., Diamandis E.P., Scarisbrick I.A. (2015). Prognostic significance of multiple kallikreins in high-grade astrocytoma. BMC Cancer.

[115] Phipps H.W., Longo L.M., Blaber S.I., Blaber M., Vanlandingham J.W. (2013). Kallikrein-related peptidase 6, a biomarker for traumatic brain injury in the rat. Brain Inj..

[116] Gaetani L., Blennow K., Calabresi P., Di Filippo M., Parnetti L., Zetterberg H. (2019). Neurofilament light chain as a biomarker in neurological disorders. J. Neurol. Neurosurg. Psychiatry.

[117] Evered L., Silbert B., Scott D.A., Zetterberg H., Blennow K. (2018). Association of Changes in Plasma Neurofilament Light and Tau Levels With Anesthesia and Surgery: Results From the CAPACITY and ARCADIAN Studies. JAMA Neurol..

[118] Bacioglu M., Maia Luis F., Preische O., Schelle J., Apel A., Kaeser Stephan A., Schweighauser M., Eninger T., Lambert M., Pilotto A. (2016). Neurofilament Light Chain in Blood and CSF as Marker of Disease Progression in Mouse Models and in Neurodegenerative Diseases. Neuron.

[119] Sutter R., Hert L., De Marchis G.M., Twerenbold R., Kappos L., Naegelin Y., Kuster G.M., Benkert P., Jost J., Maceski A.M. (2020). Serum Neurofilament Light Chain Levels in the Intensive Care Unit: Comparison between Severely Ill Patients with and without Coronavirus Disease 2019. Ann. Neurol..

[120] Garcia M.A., Barreras P.V., Lewis A., Pinilla G., Sokoll L.J., Kickler T., Mostafa H., Caturegli M., Moghekar A., Fitzgerald K.C. (2021). Cerebrospinal fluid in COVID-19 neurological complications: Neuroaxonal damage, anti-SARS-Cov2 antibodies but no evidence of cytokine storm. J. Neurol. Sci..

[121] Paterson R.W., Benjamin L.A., Mehta P.R., Brown R.L., Athauda D., Ashton N.J., Leckey C.A., Ziff O.J., Heaney J., Heslegrave A.J. (2021). Serum and cerebrospinal fluid biomarker profiles in acute SARS-CoV-2-associated neurological syndromes. Brain Commun..

[122] Ceylan U., Haupeltshofer S., Kämper L., Dann J., Ambrosius B., Gold R., Faissner S. (2021). Clozapine Regulates Microglia and Is Effective in Chronic Experimental Autoimmune Encephalomyelitis. Front. Immunol..

[123] Ehlenbach W.J., Gilmore-Bykovskyi A., Repplinger M.D., Westergaard R.P., Jacobs E.A., Kind A.J.H., Smith M. (2018). Sepsis Survivors Admitted to Skilled Nursing Facilities: Cognitive Impairment, Activities of Daily Living Dependence, and Survival. Crit Care Med..

[124] De Melo G.D., Lazarini F., Levallois S., Hautefort C., Michel V., Larrous F., Verillaud B., Aparicio C., Wagner S., Gheusi G. (2021). COVID-19-related anosmia is associated with viral persistence and inflammation in human olfactory epithelium and brain infection in hamsters. Sci. Transl Med..

[125] Von Bahr Greenwood T., Palmkvist-Kaijser K., Chiang S.C., Tesi B., Bryceson Y.T., Hjelmqvist H., Henter J.I. (2019). Elevated ferritin and soluble CD25 in critically ill patients are associated with parameters of (hyper) inflammation and lymphocyte cytotoxicity. Minerva Anestesiol..

[126] Namaste S.M., Rohner F., Huang J., Bhushan N.L., Flores-Ayala R., Kupka R., Mei Z., Rawat R., Williams A.M., Raiten D.J. (2017). Adjusting ferritin concentrations for inflammation: Biomarkers Reflecting Inflammation and Nutritional Determinants of Anemia (BRINDA) project. Am. J. Clin. Nutr..

[127] Cheng L., Li H., Li L., Liu C., Yan S., Chen H., Li Y. (2020). Ferritin in the coronavirus disease 2019 (COVID-19): A systematic review and meta-analysis. J. Clin. Lab. Anal..

[128] Singh R., Hemati H., Bajpai M., Yadav P., Maheshwari A., Kumar S., Agrawal S., Sevak J.K., Islam M., Mars J.S. (2021). Sustained expression of inflammatory monocytes and activated T cells in COVID-19 patients and recovered convalescent plasma donors. Immun Inflamm Dis..

[129] Abdelmoaty M., Yeapuri P., Machhi J., Olson K., Shahjin F., Zhou Y., Jingjing L., Pandey K., Acharya A., Byrareddy S. (2021). Defining the Immune Responses for SARS-CoV-2-Human Macrophage Interactions. bioRxiv.

[130] Trombetta A.C., Farias G.B., Gomes A.M.C., Godinho-Santos A., Rosmaninho P., Conceição C.M., Laia J., Santos D.F., Almeida A.R.M., Mota C. (2021). Severe COVID-19 Recovery Is Associated with Timely Acquisition of a Myeloid Cell Immune-Regulatory Phenotype. Front. Immunol..

[131] Zhang D.-Q., Wang R., Li T., Zhou J.-P., Chang G.-Q., Zhao N., Yang L.-N., Zhai H., Yang L. (2016). Reduced soluble RAGE is associated with disease severity of axonal Guillain-Barré syndrome. Sci. Rep..

[132] Shang P., Feng J., Wu W., Zhang H.-L. (2021). Intensive Care and Treatment of Severe Guillain-Barré Syndrome. Front. Pharmacol..

[133] Lu M.O., Zhu J. (2011). The role of cytokines in Guillain-Barré syndrome. J. Neurol..

[134] Plummer S., Van den Heuvel C., Thornton E., Corrigan F., Cappai R. (2016). The Neuroprotective Properties of the Amyloid Precursor Protein Following Traumatic Brain Injury. Aging Dis..

[135] Lue L.-F., Guerra A., Walker D.G. (2017). Amyloid Beta and Tau as Alzheimer’s Disease Blood Biomarkers: Promise from New Technologies. Neurol. Ther..

[136] Humphreys D.T., Carver J.A., Easterbrook-Smith S.B., Wilson M.R. (1999). Clusterin Has Chaperone-like Activity Similar to That of Small Heat Shock Proteins. J. Biol. Chem..

[137] Desikan R.S., Thompson W.K., Holland D., Hess C.P., Brewer J.B., Zetterberg H., Blennow K., Andreassen O.A., McEvoy L.K., Hyman B.T. (2014). The Role of Clusterin in Amyloid-β–Associated Neurodegeneration. JAMA Neurol..

[138] Götte M. (2021). Remdesivir for the treatment of Covid-19, the value of biochemical studies. Curr. Opin. Virol..

[139] Acosta-Ampudia Y., Monsalve D.M., Rojas M., Rodríguez Y., Gallo J.E., Salazar-Uribe J.C., Santander M.J., Cala M.P., Zapata W., Zapata M.I. (2021). COVID-19 convalescent plasma composition and immunological effects in severe patients. J. Autoimmun..

[140] Quintas-Neves M. (2021). COVID-19 Related Central Nervous System Vasculopathy: Beyond Vasculitis. AJNR Am. J. Neuroradiol..

[141] Rathcke C.N., Vestergaard H. (2009). YKL-40—An emerging biomarker in cardiovascular disease and diabetes. Cardiovasc. Diabetol..

[142] Kjaergaard A.D., Johansen J.S., Bojesen S.E., Nordestgaard B.G. (2015). Elevated Plasma YKL-40, Lipids and Lipoproteins, and Ischemic Vascular Disease in the General Population. Stroke.

[143] Guo X., Qiu W., Garcia-Milian R., Lin X., Zhang Y., Cao Y., Tan Y., Wang Z., Shi J., Wang J. (2017). Genome-wide significant, replicated and functional risk variants for Alzheimer’s disease. J. Neural Transm..

[144] Zavala S., Larson J., O’Mahony M., Rech M.A. (2020). Impact of insufficient admission vitamin D serum concentrations on sepsis incidence and clinical outcomes in patients with thermal injury. Burns.

[145] Yoo J.W., Kim R.B., Ju S., Lee S.J., Cho Y.J., Jeong Y.Y., Lee J.D., Kim H.C. (2020). Clinical Impact of Supplementation of Vitamins B1 and C on Patients with Sepsis-Related Acute Respiratory Distress Syndrome. Tuberc. Respir. Dis..

[146] Zhou W., Mao S., Wu L., Yu J. (2018). Association Between Vitamin D Status and Sepsis. Clin. Lab..

[147] Laudanski K. (2021). Persistence of alterations in lipoproteins and cholesterol during and after septic episode—Review of current evidence of long-term post septic lipid profile aberrations and their impli-cation for allostasis. Int. J. Mol. Sci..

