# Supplementary material for: Dynamic Changes in Central and Peripheral Neuro-Injury vs. Neuroprotective Serum Markers in COVID-19 Are Modulated by Different Types of Anti-Viral Treatments but Do Not Affect the Incidence of Late and Early Strokes
Source: Biomedicines. 2021 Nov 29;9(12):1791. doi: 10.3390/biomedicines9121791 (PMC8698659; doi:10.3390/biomedicines9121791)
Supplement: Supplementary file 1 [file biomedicines-09-01791-s001.zip › biomedicines-1379779-supplementary.pdf]

Supplemental Figures.

Supplemental Figure S1. The distribution of serum samples from admission.

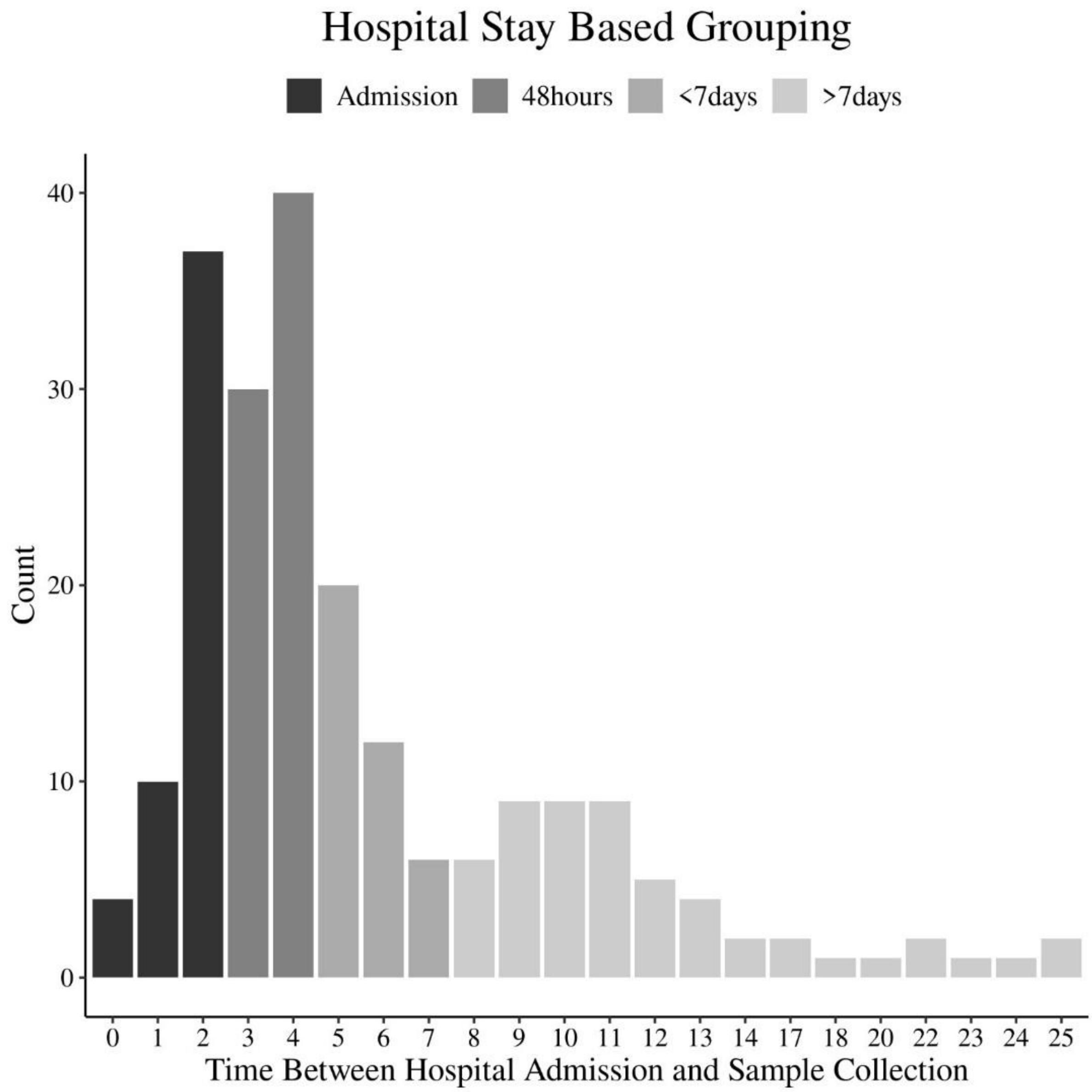

Supplemental Figure S2. Patient disposition at one- and six-months post-hospital admission.

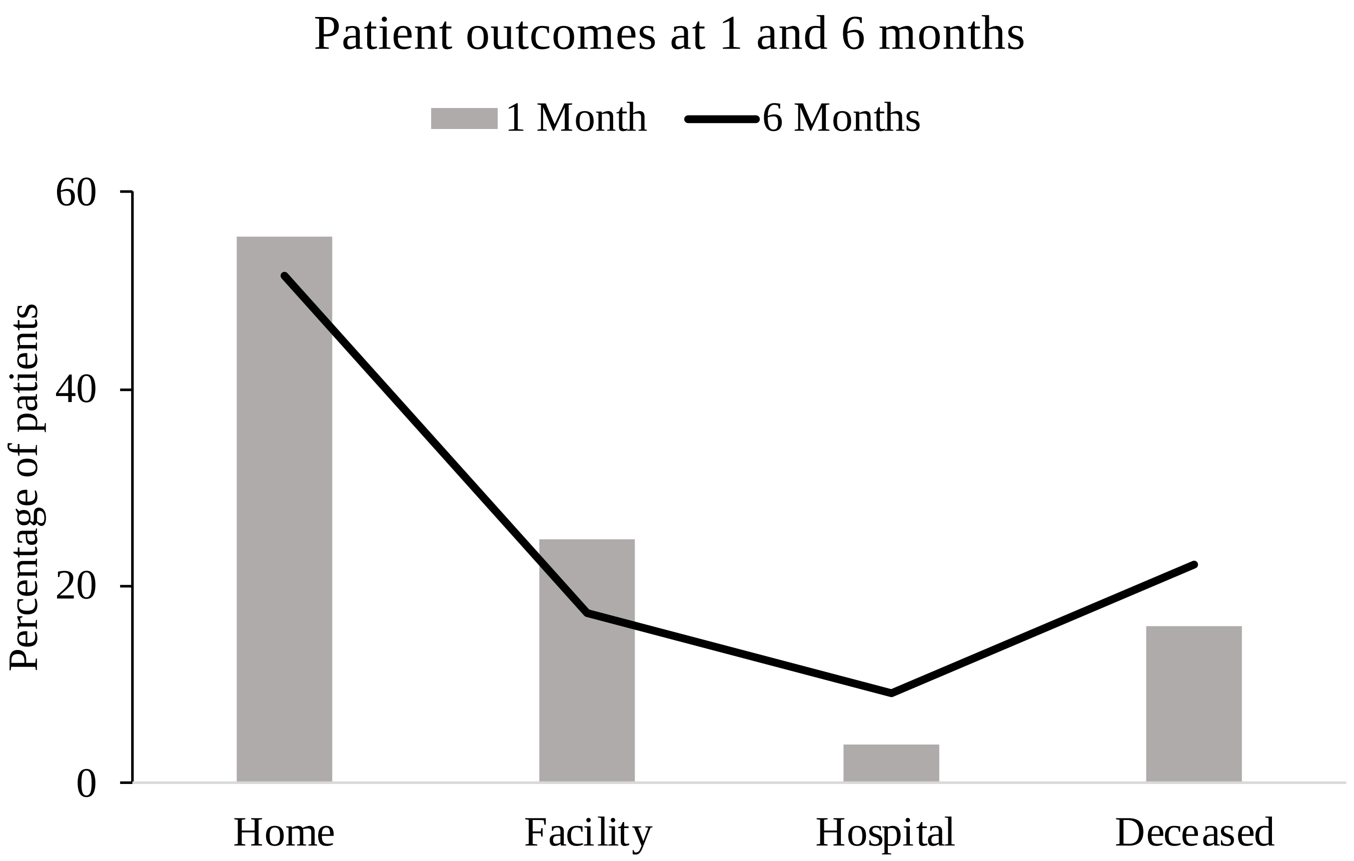

Supplemental Figure S3. Serum levels of phosphorylated tau (A) and total tau (B) were highly variable.

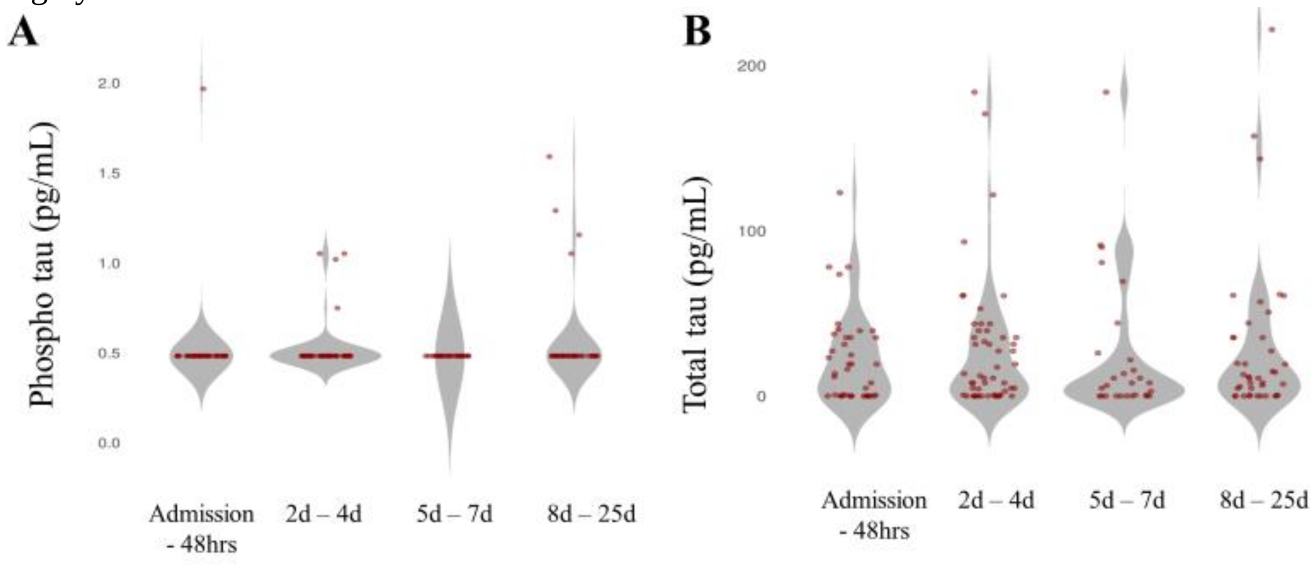

Supplemental Figure S4. Correlations between viral burden, COVID-19 direct immune system response, and neurodegeneration markers at admission (A) and throughout patient stay (B).

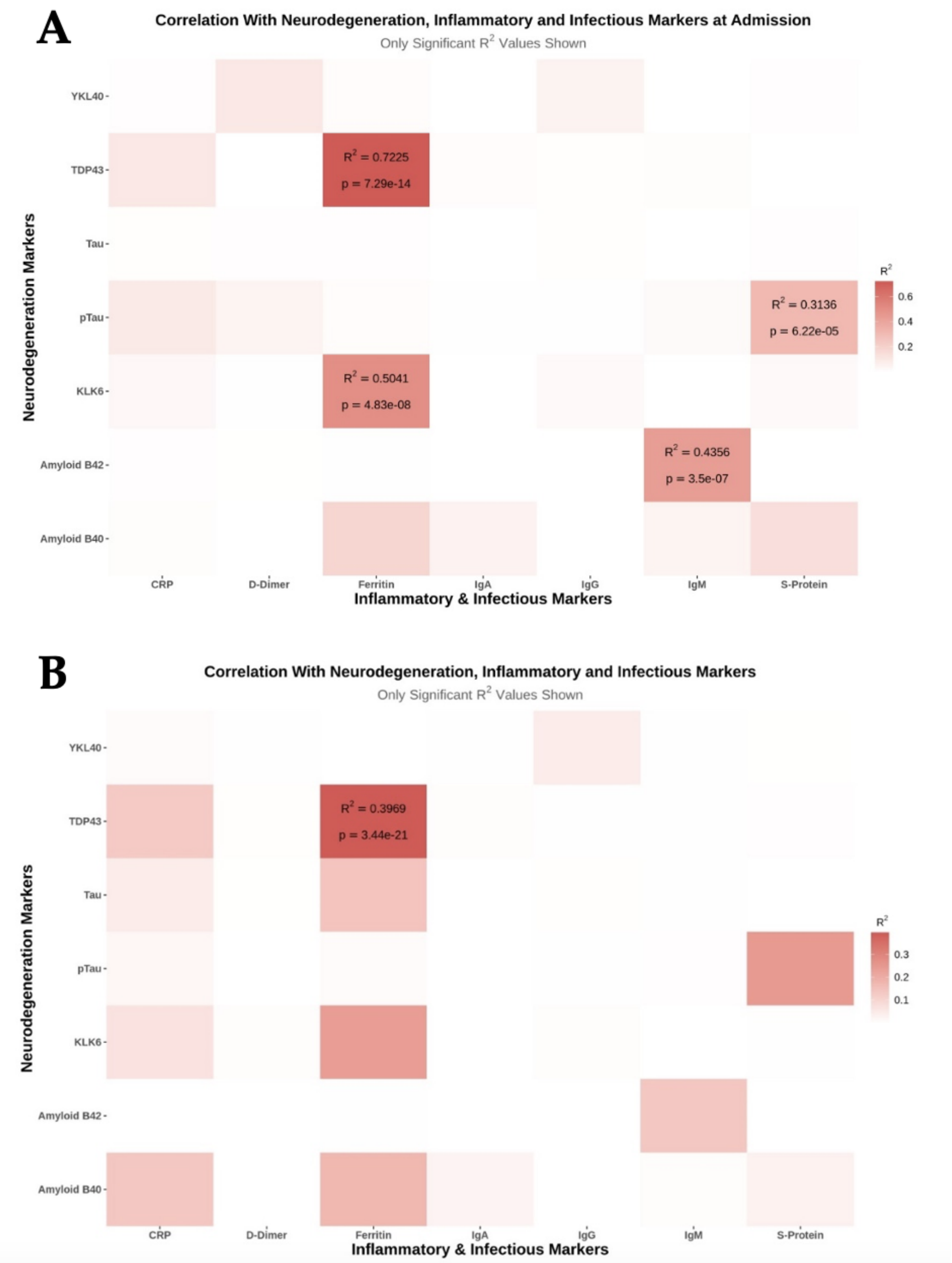

Supplemental Table S1. Comparison of markers between all patients, and patients with *vs*. without stroke.

D-dimer		Admission – 48hours	2d – 4d	2d – 4d	8-25days
	All	705.1±1078.04	1147.1±1829.87	10519.6±1869.23	3147.6±4184.11
	Stroke	218.7±7710.29	113.6±124.34	141.3±244.34	**8974.2±2128.97**[&&]
	No Stroke	726.7±2506.73	1281.1±6655.169	11672.7±5581.85	**1735.1±3575.42**[&]
Procalcitonin		Admission – 48hours	2d – 4d	2d – 4d	8-25days
	All	3.8±5.99	3.1±3.93	**1.8±1.27***[*]	**1.4±1.25**[*]
	Stroke	0.8±0.65	15±0.11	4.9±N/A	0.7±N/A
	No Stroke	4.1±11.49	3.6±5.3	**1.8±1.62**[#]	**1.6±1.85**[#]
SOFA		Admission – 48hours	2d – 4d	2d – 4d	8-25days
	All	3.1±3.26	2.6±2.73	3.3±3.28	**4.2±4.24**[*]
	Stroke	5.3±3.71	**2.9±2.33**[##]	**4.6±5.13**[#&]	3.6±3.51
	No Stroke	2.9±3.15	2.5±2.78	**3.1±2.92**[#&]	**4.4±3.66**[#]

*Statistical difference when compared to all patients at admission (p<0.039).
#Statistical difference when compared to patients who did not have a stroke at admission (p<0.018).
##Statistical difference when compared to patients who did have a stroke at admission (p<0.010).
&Statistical difference when comparing patients who did *vs* did not have a stroke at a given timepoint (p<0.041).
